# 

**Published:** 1852-09

**Authors:** 


### THE
### WESTERN JOURNAL
### OF
### MEDICINE AND SURGERY:

EDITED BY

LUNSFORD P. YANDELL, M. D.,

PROFESSOR OF PHYSIOLOGY AND PATHOLOGICAL ANATOMY IN THE UNIVERSITY OF LOUISVILLE,

AND

THEODORE S. BELL, M. D.

CXLXIII.—SEPTEMBER, 1852.

**THIRD SERIES.**
Vol. X......No. III

LOUISVILLE, KY:
PUBLISHED MONTHLY BY PRENTICE & HENDERSON,
PROPRIETORS AND PRINTERS.
1852.

